# Looking Out for The Secret Wound: The Effect of E-Cognitive Group Therapy with Emotional Disclosure on The Status of Mental Health in Infertile Women

**Published:** 2012-09-20

**Authors:** Leili Mosalanejad, Anahita Khodabakhshi Koolaee, Bahar Morshed Behbahani

**Affiliations:** 1. Jahrom University of Medical Sciences, Jahrom, Iran; 2. Department of Psychology, University of Social Welfare and Rehabilitation Science, Tehran, Iran; 3. Department of Midwifery, Shiraz University of Medical Sciences, Shiraz, Iran

**Keywords:** Assisted Reproductive Therapy, Cognitive Behavior Therapy, Mental Health, Infertility

## Abstract

**Background:**

Considering the high prevalence of psychiatric disorders among infertile women, it seems that gynecologists, psychiatrists, and psychologists should be more attentive to identify and treat these disorders. The aim of this study is to determine the effect of E-cognitive group therapy with emotional disclosure on mentwal health status of infertile women who are receiving assisted reproduction.

**Materials and Methods:**

In this randomized clinical trial study, 80 infertile women who were receiving hormonal therapy or other assisted reproductive technologies (ART) were randomly allocated to the cognitive-behavioral treatment (CBT) group or the control group. The CBT group had a weekly 12-hour meeting for a period of three months. They also participated in some painting sessions (art therapy) and written and verbal emotional disclosure (both individually and in group presentation). The Depression, Anxiety, Stress Scales (DASS) test and Penn State Worry Questionnaire (PSWQ) were used for data gathering.

**Results:**

Results showed the level of psychological distress decreased in the control group, but not significantly. Psychological intervention in the treatment group significantly lowered the level of psychological distress; the mean score of DASS in all aspects was significant. The difference between the mean score of the two groups after intervention was significant (p=0.001) and also according to ANCOVA (p=0.002). Differences were significant between the mean scores of both groups in the PSWQ (p=0.001), Inventory Test (p=0.001), which was confirmed by ANCOVA (p=0.009).

**Conclusion:**

These finding suggest that CBT with emotional self-disclosure promotes coping strategies among infertile women. Results also show that these approaches develop mental health and decrease stress in infertile women. Using a psychiatric approach in medical settings could help infertile women to promote their adjustment with mental health problems due to of in infertility. (Registration Number: IRCT201108247407N2).

## Introduction

Pregnancy and childbirth are typically associated with positive emotions. Evidence suggests that the anxiety and depression associated with infertility are similar to those associated with other serious medical conditions, such as cancer and human immunodeficiency virus (HIV) (1). Infertility is a major medical problem that affects many couples (2). About 10%- 15% of childbearing-age couples experience infertility, which can affect them physically, emotionally, financially, socially, and psychologically (3).

The psychological aspect of infertility is much more difficult diagnose and treat. This is one of the greatest issues for many couples (4-6). The recognition of the painful character of infertility diagnosis and treatment has led to the development of several psychosocial interventions for infertile couples. Psychological treatments include psychotherapy and cognitive behavioral therapy (CBT), and can diminish several mental disorders, such as phobia, depression, and anxiety. They can also enhance one’s physical health, thus resulting in a successful pregnancy (7).

Infertility should be considered a bio-psychosocial crisis, with psychological counseling being an integral part of a multidimensional solution (8). A decrease in the level of depression, anxiety, mental distress, and rate of pregnancy have been reported in some studies following psychosocial interventions (9, 10). Many studies have assessed other dimensions as well, including self-efficacy, implantation rate, desire for a child, and the positive impact of psychological interventions on pregnancy rates (9, 11-13).

It is a chance for clinical specialists in the field of mental health to treat couples dealing with infertility (14).

Due to the high prevalence of psychiatric disorders in infertile women, doctors, psychiatrists, and psychologists should look more carefully for both early detection and treatment of these disorders (15). According to the emotional distress as a consequence of infertility and the problems in treating them, several studies have recommended psychological intervention for infertile couples. However couples, themselves, sometimes wish obtain additional psychosocial support (16, 17). The aim of this study is to assess the impact of group behavioral therapy with emotional disclosure on infertile psychological stress (anxiety-depression-stress) and infertility concerns.

## Materials and Methods

### Participants

This is a randomized clinical trial on 80 infertile women chosen by simple sampling in the Jahrom Gynecology Clinic, affiliated with Jahrom University of Medical Sciences.

Infertility was defined as at least one year of unprotected coitus without conception. Women who met the inclusion criteria were recruited to our study. All participants who had primary infertility, with no somatic and psychiatric problems, who were residents of Jahrom, ages 20-40 years, had a valid cell phone number, were able to read and write, and who were interested in participating in regular group meetings were selected for our study. All patients signed a consent form to participate in our study and there was no obligation for them to participate in this research. The Institutional Ethics Committee approved the study protocol.

A total of 80 infertile women consecutively included in infertility therapy or other ART were randomly allocated either to the cognitive-behavioral treatment (CBT) group or to the control group.

In the first two sessions, 16 patients were absent and some did not complete the entire questionnaire, therefore the study continued with two groups of 32 patients in the CBT group and 33 in the control group. 12 meetings were provided for a period of 3 months, as a 2 hour weekly meeting.

### Cognitive-behavioral treatment group meeting

The group therapy used in this study addressed causes of infertility and aimed to teach participants how to recognize and challenge negative thoughts and irrational beliefs. Also CBT group therapy, included cognitive reconstructing, negative thought blocking, spirituality (finding the meaning of life in the heart of the problem), and the behavioral techniques included muscle relaxation, birth exercises, imagination(replacing negative thoughts with positive thoughts in mind), self-disclosure and biofeedback in group, 2 hours weekly to 12 sessions. All of participants received positive massages about cognitive reconstructing; changing negative thoughts and how to induce positive thoughts about their infertility and brief messages regarding the results of the session. All participants had emotional disclosure in 30 sessions. Participants expressed positive or negative feelings about their problems and their peers could give them feedback. In some sessions, participants painted their feelings by means of art therapy for emotional disclosure. All paintings were analyzed by expert specialists and participants individually received feedback from group leaders about their emotions. Participants also presented positive and negative emotional feelings about their problems as a written task or expressed them in a group.

At the beginning of each session and in two additional sessions, we used interactive animation videos about the infertility medical approach and specialists responded to personal questions about the infertility interventions. Table 1 is refers to contents and duration of interventions.

**Table 1:** The contents of interventions among three psychiatric approaches


Psychiatric approach	Subjects	Duration

**CBT group**	Cognitive reconstructing, stress reductionin group	10 sessions
**Emotional disclosure**	Decreasing negative thoughts and feelings,expressing them by various methods	12 sessions
**Interactive multimedia video**	Giving information, stress reduction,organizing treatment plan	2 sessions


**Table 2:** Differences in mean scores of Depression Anxiety Stress Scales (DASS) test within groups before and after intervention


	DASS	Pre-test	Post-test	T	P

**Treatment group**	Anxiety	13.96(2.59)	8.06(2.63)	8.40	0.000
	Depression	14 (2.38)	8 (2.62)	10.33	0.000
	Stress	13.93(3.15)	8.84(2.65)	13.19	0.000
	Total	41.90 (6.49)	8.84(2.65)	32.67	0.000
**Control group**	Anxiety	9.41(3.45)	9.25(3.26)	0.29	0.77
	Depression	8.87(3.54)	7.80(3.15)	2.50	0.01
	Stress	11.93 (3.74)	11.41 (3.21)	0.93	0.35
	Total	30.22 (3.22)	29.89 (3.23)	0.22	0.87


The Depression Anxiety Stress Scales (DASS) test was used to collect data. This test is the shortform of the DASS-21, which served as a standard reference. The DASS-21 is a 21-item exam designed to measure the three negative affective states of depression, anxiety, and stress. According to Lovibond and Lovibond, "The depression scale assesses dysphoria, hopelessness, devaluation of life, self-deprecation, and lack of interest or involvement, anhedonia and inertia. The anxiety scale assesses autonomic arousal, situational anxiety, and subjective experience of anxious affect. The stress scale assesses difficulty relaxing, nervous arousal, and being easily upset or agitated, irritable, or over-reactive and impatience." (18). Using the cutoff scores suggested by Lovibond and Lovibond (19, 20), the psychometric properties of the DASS have been extensively evaluated, and there is evidence for the convergent and discriminative validity of data obtained with the instrument (18, 21-23).

The Penn State Worry Questionnaire (PSWQ) was used in both clinical and non-clinical situations (3). According to Brown et al. "This tool consisted of 16 Likert items and showed excellent internal consistency, test-retest reliability, and concurrent and discriminative validities.In a clinical group study by, the PSWQ showed high reliability and validity as well" (24).

### Statistical analysis

Data were analyzed by SPSS statistical software version-18 (SPSS Inc; Chicago, IL) for analyzed the demographics characteristics applied descriptive statistics. Also, for assessed the hypothesis of research used the paired t test to compare mean of variation in two groups, and analysis of covariance (ANCOVA) to compare means of variation between two groups.

Informed consent was taken from patients and the study protocol was approved by the Institutional Ethics Committee of Jahrom University of Medical Sciences.

### Results

The current study included 33 infertile women in the treatment group and 32 infertile women in the control group. The demographic characteristics of the study groups showed no significant difference between the treatment and control groups for age (p=0.43), education (p=0.13), duration of infertility, and etiology of infertility (p=0.26).

The age of women ranged from 20-30 years in 18 cases in the treatment group and 31-40 years in 19 patients in the control group (p=0.43). The highest range was 19 (59.4%) in the treatment group and 18 (58.1%) in the control group.

The education level was similar in the treatment and control groups (p=0.13). There were 62 patients (81.25%) in both groups that had a diploma while 18 patients (21.9%) had secondary education or less.

Mean infertility duration in the treatment group was 12 years and in the control group was 16 years; there was no significant difference between the groups (p=1).

According to the etiology of infertility, female factor infertility was observed in 18 patients (56.2%) in the treatment group and 16 patients (51.6%) in the control group. Male factor infertility was observed in 3 patients (9.4%) in the treatment group and 7 (22.6%) in the control group. The cause of infertility was similar in both groups (p=0.26).

Results of the DASS are presented in table 2. Compared to baseline, psychological intervention in the treatment group significantly decreased the level of psychological distress and anxiety (13.96 ± 2.59 vs. 8.06 ± 2.63); depression (14 ± 2.38 vs. 8 ± 2.62); and stress (13.93 ± 3.15) vs. 8.84 ± 2.65. Total DASS highly decreased in the treatment group 41.90 ± 6.49 vs. 8.84 ± 2.65. In the control group there was also a decreased level of psychological distress and anxiety (9.41 ± 3.45 vs. 9.25 ± 3.26); depression (8.87 ± 3.54 vs. 7.80 ± 3.15); stress (11.93 ± 3.74 vs. 11.41 ± 3.21). However none were significant.

There was a significant difference between the DASS mean score after intervention in the groups as the level of stress in the treatment group decreased more than the control group. This result was confirmed by the analysis of covariance test (ANCOVA; p=0.001, T=0.14; F=0.14, p=0.002; Table 3).

Other results showed psychological intervention in the treatment group significantly decreased the PSWQ score from 33.25 ± 12.24 to 27.31 ± 13.50 (p=0.004).

The PSWQ score in the control group was 34.19 ± 8.80 at baseline and 34.45 ± 8.23 at the end of the study. This difference was not significant (p=0.65; Table 4).

Difference of the mean score of the PSWQ in two groups was significant after intervention (p=0.01) and this result was confirmed by ANCOVA (p= 0.009; Table 5).

**Table 3:** Differences in mean Dpression Anxiety Stress Scales (DASS) score between groups after intervention


DASS	Treatment group	Control group	T	F	P	

**Anxiety**	8.06 (2.63)	9.25(3.26)	1.48	2.94	0.11*	0.09**
**Depression**	8 (2.62)	7.80(3.15)	1.18	000	0.76*	0.99**
**Stress**	8.84 (2.65)	11.41 (3.21)	0.14	10.32	0.001***	002***
**Total**	23.93 (6.78)	26.62 (7.61)	0.12	2.07	0.15*	0.15**


* Independent t test, ** ANCOVA test, *** p=<0.05.

**Table 4:** Differences in mean score of Penn State Worry Questionnaire (PSWQ) within groups before and after intervention


Group	Pre-test	Post-test	T	P

**Treatment group**	33.25(12.24)	27.31(13.50)	3.06	0.004
**Control group**	34.19(8.80)	34.45(8.23)	-0.45	0.65


* Independent t test, ** ANCOVA test, *** p=<0.05.

**Table 5:** Differences between mean score of Penn State WorryQuestionnaire (PSWQ) between groups


State	Group	Mean (SD)	T	F	P	

**Pre-test**	Experiment	33.25(12.24)	5.01	0.27	0.72*	0.60**
	Control	34.19(8.80)				
**Post-test**	Experiment	27.31(13.50)	11.37	7.28	0.01 ***	0.009***
	Control	34.45 (8.23)				


* t test, ** ANCOVA, *** p=<0.05.

## Discussion

The current study showed that using a psychological approach such as a CBT or E cognitive message with self-disclosure has a significant impact on psychological distress and improved infertile women’s mental health.

Many studies have investigated the effect of the psychiatric approach on infertile women. Since new methods are constantly being introduced in the field of infertility, the psychological issue has recently become an important issue that needs to be addressed (25).

In one study, it has been reported that providing information about the technical aspects of infertility treatment can facilitate coping with infertility and medical treatments. This information can be given from booklets, interactive films, and multimedia. Although the internet is the fastest and easiest way to obtain information on infertility and related treatments, there is the possibility of wrong or misleading information.

Telephone counseling can be useful in providing specific information about infertility, but in difficult psychological issues it is not a substitute for face-to-face counseling (26).

Studies have confirmed our results, showing the impact of interactive multimedia on infertile psychological distress. Multimedia methods may act as a novel and real intervention for infertile patients, which can be a solution in cases of limited local access to educational and support services (27).

We used paintings as a method of expressing feelings. Some research has shown the effect of this method in lowering the negative emotions of infertile women.

Art therapy is an inexpensive, non-pharmacological intervention associated with decreased levels of hopelessness and depression in subfertile women. It also provides insight into the meaning and emotional implications of subfertility for patients and caregivers (28).

Other studies state that self-psychology therapy provides a valuable framework for the therapist, given the profound and multiple narcissistic assaults on self-esteem, consolidation of identity, developmental aspirations, and other self attributes which infertility causes. The therapist’s empathy becomes the primary tool of both understanding and alleviating suffering resulting from infertility (29). More studies are needed to confirm these results.

Using multimedia and the internet significantly reduced the depression level of clinically distressed and depressed participants. Internet-based interventions have been show to be promising new approaches for infertile patients (30). The treatment was assessed as positive or very positive by 80% of the participants. However, further research is needed.

In many studies, cognitive behavioral approaches have been shown to be effective for individuals dealing with anxiety and assist infertile patients in challenging and coping with anxiety.

While some research findings have demonstrated that psychiatric interventions (behavioral, cognitive, psychotherapy) increase the rate of pregnancy, others have not found improved pregnancy rates, but noted decreased rates of depression and anxiety (31, 32).

In the absence of clinical and mental disorders, the use of psychological approaches can increase chances of pregnancy in patients. Using a psychological approach is a proper treatment, especially for those who are not receiving medical treatment (33).

Stress management is an effective treatment and should be offered to patients before, during, and after undergoing the additional stress of assisted conception treatments (34).

Some studies have stated that both psychotherapy and CBT are well-established treatments for depression and anxiety (35). Tarabusi et al. (31) and Kupka et al. (36) have shown that CBT avoids 'waiting stress' that could be useful for stimulating discussion and awareness for the couple.

Several studies have demonstrated the importance of the mind–body connection and fertility (36). Domar et al. (33), Terzioglu (34) and Place et al. (37) have reported that psychiatric interventions led to significant decreases in anxiety and depression and an increased chance of pregnancy.

Some studies have shown that various psychological treatments can make a contribution to lower stress, but they rarely increase the possibility of pregnancy (38).

Pakenham and Rinaldis (39) and Hart (40). have reported that psychological intervention in 14% of cases is conducive to spontaneous pregnancy and that it can be a consequence of reduced stress (40, 41).

In our study there was a wide age range of between 20-40 years. This might have confessional result, as women in their 40s may have more depression compared with women in their 20s because of the possibility of being infertile for a longer time.

Additional factors such as women’s social skills and their perception of their own mothers’ acceptance or rejection could cause depression (41). These factors and the effect of e-cognitive group therapy with emotional disclosure should be addressed in future studies. We suggest that the first clinical impressions about the usefulness of the body-mind group program in fertility clinics seems to be promising. Further research is needed to assess its effectiveness.

## Conclusion

Typically, group therapy involves a group of individuals with a similar struggle, sitting together and talking about their lives and concerns under the guidance of a certified counselor. According to the results and in attention to the effect of this approach to infertile psychological distress, we suggest psychological approaches associated with other new methods which can help couples to handle their problem. This approach can also help to improve mental health status in infertile women.
